# Bloodstream Infections and Antimicrobial Resistance in The Gambia: Continuous Surveillance from a Tertiary Care Center

**DOI:** 10.4269/ajtmh.25-0199

**Published:** 2025-09-23

**Authors:** David Nygren, Felix Andersson, Ebrima Barrow, Sheikh Omar Bittaye, Haddy Bah, Fatou Banja, Kumba Suun Mboob, Lamin Fatajo, Marisel Gomez Blanco, Emmanuel Olabode, Alieu Jallow, Abdoulie Badjan, Paul Rahden

**Affiliations:** ^1^ Department of Infectious Diseases, Skåne University Hospital, Malmö, Sweden;; ^2^ Division of Infection Medicine, Lund University, Lund, Sweden;; ^3^ Microbiology Unit, Department of Laboratory Medicine, Edward Francis Small Teaching Hospital, Banjul, The Gambia;; ^4^ Department of Internal Medicine, Edward Francis Small Teaching Hospital, Banjul, The Gambia;; ^5^ School of Medicine and Allied Health Sciences, University of The Gambia, Banjul, The Gambia;; ^6^ RG Neglected Diseases and Envenoming, Bernhard Nocht Institute for Tropical Medicine, Hamburg, Germany

## Abstract

Antimicrobial resistance (AMR) poses a major threat to global health, with limited surveillance data available from western sub-Saharan Africa. After reports of high rates of methicillin-resistant *Staphylococcus aureus* (MRSA) and extended-spectrum beta-lactamase (ESBL) in bloodstream infections at The Gambia’s sole tertiary hospital, we present follow-up data after enhancements in microbiology capacity. This study included 1,010 patients with blood cultures taken at Edward Francis Small Teaching Hospital between September 2023 and August 2024. The positivity rate remained high (31%), particularly among neonates and critically ill patients. *Staphylococcus aureus* was the most frequently isolated pathogen (49%, *n *= 155/314), with MRSA identified in 22% (*n *= 34/152) of tested isolates. Among Enterobacterales, ESBL production remained high (87%, *n *= 84/97), and carbapenem resistance was detected in 15% (*n *= 6/39) of tested isolates. Our findings highlight the need for a robust and sustained AMR surveillance system to inform targeted interventions aimed at reducing the emergence and spread of multidrug-resistant pathogens.

## INTRODUCTION

Antimicrobial resistance (AMR) is a major threat to public health globally; 1.27 million attributable deaths were estimated in 2019, of which 250,000 occurred in Africa alone,^1^ with western sub-Saharan Africa particularly affected.^2^ *Klebsiella pneumoniae *resistant to third-generation cephalosporins (extended-spectrum beta-lactamase [ESBL]) and methicillin-resistant *Staphylococcus aureus* (MRSA) have been estimated to be the leading pathogen–drug combinations causing death attributable to AMR. However, bacteriological diagnostics, antimicrobial susceptibility testing (AST), and surveillance data remain scarce in western sub-Saharan Africa, adding uncertainty to the interpretation of available data.^2^^–^^4^

Previous reports on bloodstream infections from our study setting, The Gambia, are few and have mainly been conducted at the Medical Research Council, The Gambia. Here, *S. aureus* was the most common cause of bacteremia,^5^^–^^7^ yet MRSA rates were low (0–2.4%), whereas ESBL rates among Enterobacterales have been reported to be as high as 35%.^5^^,^^6^^,^^8^^,^^9^ Recently, we presented surveillance data from the only tertiary hospital in The Gambia: Edward Francis Small Teaching Hospital (EFSTH).^10^ In contrast to previous findings, we identified a majority of *S. aureus* to be MRSA (59%) and described an ESBL rate of 90% among Enterobacterales. The blood culture positivity rate was high (40%), and a majority of patients were cultured in an intensive care unit (ICU; 63%), indicating the presence of selection bias of severely ill patients as well as potentially, patients already failing first-line therapy.^10^ In addition, the EFSTH represents the main referral hospital in the country, resulting in a further selected patient clientele. Consequently, the generalizability of findings is limited, yet results remain concerning. Recently, after a quality improvement program, microbiological testing capabilities and antimicrobial stewardship initiatives have been supported at the EFSTH. The primary hypothesis of this study was that with increased testing availability, the blood culture positivity and AMR rates would decrease given the lower impact of selection bias. Thus, the objective was to investigate species distribution and AMR after increased availability and use of microbiological diagnostics.

## MATERIALS AND METHODS

This was an observational, retrospective study investigating blood culture findings at the EFSTH in Banjul, the sole tertiary hospital in The Gambia. It serves as the only referral center offering specialist care, dialysis, and intensive care in the country. All patients with blood cultures drawn at the EFSTH from September 2023 to August 2024 were included. The data were collected from microbiological records at the Microbiology Unit and included the date of the culture, age, sex, hospital ward, microbiological finding, and AST. No clinical data were available. Patients were categorized in three age groups; neonates (defined as 28 days old or younger), pediatric (older than 28 days old to younger than 15 years old), and adults (15 years old or older). If age data were missing, patients were categorized based on the ward where the samples were drawn.

Blood cultures were collected according to local guidelines using two BD BACTEC PLUS (bd.com) culture bottles for adults, one aerobic and one anaerobic, whereas in pediatric and neonatal patients, one BD BACTEC PLUS pediatric culture bottle was drawn. Bottles were incubated as described elsewhere.^10^ Anaerobic incubation was not performed as part of routine practice. Species identification was performed using gram staining, basic biochemical testing, and assessment of morphological appearance as described previously.^10^ Antimicrobial susceptibility testing was performed according to the Kirby–Bauer disc diffusion method using Clinical and Laboratory Standards Institute break points.

Reporting of AST data focused on the presence of MRSA, ESBL, ESBL carbapenemases, and acquired resistance among *Pseudomonas aeruginosa*. To be classified as MRSA, *S. aureus* isolates were required to demonstrate cefoxitin resistance or resistance to any other normally *S. aureus*-active beta-lactam antibiotic if cefoxitin testing was not available. All other strains were classified as methicillin-susceptible *S. aureus* if AST was performed.

Extended-spectrum beta-lactamase was defined as resistance to third-generation cephalosporins or broader in pathogens of the Enterobacterales group using double-disc synergy testing. Isolates evaluated for carbapenem resistance were reported separately. Acquired resistance in *P. aeruginosa* was defined as any acquired resistance beyond wild-type susceptibility defined as any resistance to ciprofloxacin, aminoglycosides (gentamicin), piperacillin-tazobactam, imipenem-cilastatin, meropenem, or ceftazidime.

Isolates registered as contaminants included coagulase-negative* Staphylococci*, *Bacillus *spp., *Micrococcus* spp., and coryneform bacteria as no clinical data were available from the microbiological records to evaluate their clinical importance.

Data were presented with descriptive statistics and reported as counts and percentages, focusing on the most common pathogens and AST overall and between age categories. In a secondary analysis, seasonal differences were assessed by comparing the wet (June to October) and dry (November to May) seasons. Statistical differences between groups in terms of blood culture positivity rates and AMR rates were assessed using the χ^2^ test or the Fisher exact test as appropriate. Statistical analyses were performed using Stata v. 17.0 (StataCorp, College Station, TX).

## RESULTS

A total of 1,010 patients were included, of which 514 (51%) were adults, 241 (24%) were pediatric patients, and 250 (25%) were neonates. Five cases lacked data on age or ward, whereas 162 of 1,010 (16%) patients lacked a registered ward. In the remaining 848 patients, 446 of 848 (53%) patients were treated in an ICU. The blood culture positivity rate was 314 of 1,010 (31%). Positivity rates were higher in neonates compared with both adults (χ^2^ *P* = 0.007) and pediatric patients (χ^2^ *P* <0.001), whereas adults had a higher positivity rate than pediatric patients (χ^2^ *P* = 0.03) (Table 1). Blood culture positivity rates were higher in ICU patients (*n *= 168/446; 37%) when compared with non-ICU patients (*n* = 95/402; 24%; χ^2^ *P* <0.001). Contaminants were found in 101 of 1,010 (10%) blood cultures.

**Table 1:** Baseline characteristics of patients across age groups, including species distribution of positive blood culture findings

Characteristics	Neonates (≤28 days; *n = *250*)	Pediatrics (>28 days to <15 years; *n = *241*)	Adults (≥15 years; *n = *514*)
Age, median (IQR)	6 (3–10) days	5 (2–9) years	27 (13–47) years
Female, % (*n/N*)	44 (110/248)	44 (105/241)	55 (285/514)
Blood culture positivity rate, % (*n/N*)	40 (101/250)	23 (55/241)	31 (157/514)
Blood culture drawn in ICU, % (*n/N*)	97 (228/235)	12 (28/225)	49 (190/388)
*Staphylococcus aureus*, % (*n/N*)^†^	38 (38/101)^‡^	58 (32/55)	54 (84/157)^§^
*Klebsiella *spp., % (*n/N*)^†^	16 (16/101)^‡^	13 (7/55)	8 (12/157)^§^
*Enterobacter *spp., % (*n/N*)^†^	11 (11/101)^‡^	7 (4/55)	9 (14/157)^§^
*Escherichia coli*, % (*n/N*)^†^	6 (6/101)^‡^	0 (0/55)	10 (15/157)^§^
Other of the Enterobacterales order, % (*n/N*)^†^	6 (6/101)^‡^	11 (6/55)	5 (8/157)^§^
*Pseudomonas aeruginosa*, % (*n/N*)^†^	16 (16/101)^‡^	6 (3/55)	9 (14/157)^§^
Other, % (*n/N*)^†^	10 (10/101)^‡^	5 (3/55)	7 (11/157)^§^

ICU = intensive care unit; IQR = interquartile range.

* Data on age category or ward status were missing in *n* = 5 samples (*n* = 5). The denominators in the respective columns represent available data as applicable.

^†^ Denominators represent the reported number of positive results in the respective age group.

^‡^ Two samples included polymicrobial growth of two pathogens.

^§^ One sample included polymicrobial growth of two pathogens.

The most common pathogens identified, in descending order, were *S. aureus* (*n* = 155/314; 49%), *Klebsiella *spp. (*n* = 35/314; 11%), *P. aeruginosa* (*n* = 33/314; 11%), *Enterobacter *spp. (*n* = 29/314; 9%), and *Escherichia coli* (*n* = 21/314; 7%). These five accounted for 273 of 314 (87%) positive cultures (Table 1). Polymicrobial infections were rare (*n* = 6). The species distribution was similar between age groups, with *S. aureus* being the most common finding in all patients (Table 1).

**Figure 1.**
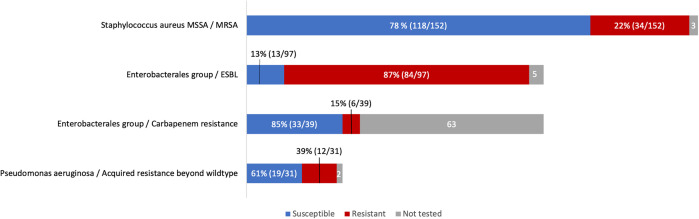
Distribution of resistance rates among common pathogens. Methicillin-resistant *Staphylococcus aureus* (MRSA) is defined as resistance to cefoxitin or other beta-lactam antibiotics if not tested for cefoxitin. Extended-spectrum beta-lactamase (ESBL) production is defined as any resistance to third-generation cephalosporins, piperacillin-tazobactam, or carbapenems. Carbapenem resistance is defined as resistance to any carbapenem. Acquired resistance beyond wild type in *Pseudomonas aeruginosa* is defined as any resistance to ciprofloxacin, aminoglycosides (gentamicin), piperacillin-tazobactam, imipenem-cilastatin, meropenem, or ceftazidime. MSSA = methicillin-susceptible *S. aureus*.

Of 155 *S. aureus* cases, 152 had AST performed, and 34 of 152 (22%) were MRSA (Figure 1). Within the Enterobacterales group, 97 of 102 (95%) had AST performed, of which 84 of 97 (87%) were ESBL. A subset (*n* = 39/102; 38%) was tested for carbapenems, with 6 of 39 (15%) found to be carbapenem resistant. Among *P. aeruginosa*, 31 of 33 (94%) were tested for resistance beyond wild-type resistance, and 12 of 31 (39%) had acquired resistance. There were no differences in MRSA rates (χ^2^ *P* = 0.45) or in acquired resistance among *P. aeruginosa* between age categories (Fisher exact *P* = 0.37); yet, for ESBL, a difference was seen (Fisher exact *P* = 0.026) because of a higher rate among adults (95%; *n* = 42/44) compared with neonates (*n* = 28/37; 76%; Fisher exact *P* = 0.02), with neonates having the lowest ESBL rate. When assessing seasonality, blood culture positivity rates were higher during the wet season (37%; *n* = 145/394) than the dry season (27%; *n* = 169/616; χ^2^ *P* = 0.002). As for species distribution, only *S. aureus* differed, and it was more commonly seen (20%; *n* = 77/394) during the wet season compared with the dry season (13%;* n* = 78/616; χ^2^ *P* = 0.003).

## DISCUSSION

We report surveillance data on bloodstream infections and AMR over a 12-month period at the sole tertiary hospital in The Gambia. Half of the patients cultured were adults, and half of the cultures were drawn in an ICU setting. A high blood culture positivity rate (31%) was found, with the most common pathogens identified being *S. aureus*, *Klebsiella* spp., *P. aeruginosa*, *Enterobacter* spp., and *E. coli*. Rates of AMR among *S. aureus* (MRSA) were higher than reported in previous studies focused on community-acquired infections in The Gambia; however, they were lower than previously reported from the same tertiary hospital, whereas ESBL rates among Enterobacterales remained very high.

This study follows a previous report from the EFSTH,^10^ where blood culture findings were investigated the preceding year. Although species distribution between studies was similar, we now found markedly lower blood culture positivity and MRSA rates. This is likely because of less restrictive testing as hypothesized, with blood cultures increasingly available and improved availability of AST with fewer stock outs. Remarkably, ESBL among Enterobacterales remained nearly ubiquitous, particularly among adult patients, suggesting a substantial presence of ESBL in the community. Additionally, carbapenem resistance was investigated in a subset and found to be present, which was not previously reported in bloodstream infections in The Gambia. Previous studies in The Gambia have mainly focused on community-acquired infections and have reported comparably low AMR rates.^5^^,^^6^^,^^8^^,^^9^ In part, our study suffers from selection bias of severely ill and referred patients because of solely including patients cultured at the EFSTH; hence, firm conclusions on presence of AMR in the community are hard to draw. However, given repeated findings of high MRSA rates and very high ESBL rates,^10^ the need for increased interventions to mitigate the impact of AMR at the EFSTH and regionally is highlighted.^1^^,^^3^ Further limitations include uncertainties associated with traditional methods in species identification, stock outs, and limited resources at the EFSTH. Currently, microbiological availability has been further expanded, and antimicrobial stewardship efforts have been implemented to address the need for increased surveillance and mitigation efforts. Future external quality control programs are being established, which include comparing traditional species identification with matrix-assisted laser desorption/ionization time of flight, and an ongoing prospective study aims to better assess causes of and AMR in community-acquired infections.

## CONCLUSION

We report surveillance data on bloodstream infections from the sole tertiary hospital in The Gambia, with *S. aureus* being the most common finding. The presence of MRSA and ESBL was found to be alarmingly high. However, severely ill patients, referred patients, and likely, patients failing first-line therapy were overrepresented, limiting generalization to community-acquired infections.
